# Comprehensive Genome-Wide Identification of the RNA-Binding Glycine-Rich Gene Family and Expression Profiling under Abiotic Stress in *Brassica oleracea*

**DOI:** 10.3390/plants12213706

**Published:** 2023-10-27

**Authors:** Mengmeng Duan, Mei Zong, Ning Guo, Shuo Han, Guixiang Wang, Liming Miao, Fan Liu

**Affiliations:** 1 Beijing Vegetable Research Center, Beijing Academy of Agriculture and Forestry Sciences, State Key Laboratory of Vegetable Biobreeding, National Engineering Research Center for Vegetables, Key Laboratory of Biology and Genetic Improvement of Horticultural Crops (North China), Ministry of Agriculture, Beijing 100097, China; duanmengmeng@nercv.org (M.D.); zongmei@nercv.org (M.Z.); guoning@nercv.org (N.G.); hanshuo@nercv.org (S.H.); wangguixiang@nercv.org (G.W.); 2 Horticulture Research Institute, Shanghai Academy of Agricultural Sciences, Shanghai 201403, China; 11616010@zju.edu.cn

**Keywords:** *Brassica oleracea*, RNA-binding glycine-rich (RBG) genes, abiotic stress, expression profiling, *BoiRBGA13*

## Abstract

The RNA-binding glycine-rich proteins (RBGs) of the glycine-rich protein family play vital roles in regulating gene expression both at the transcriptional and post-transcriptional levels. However, the members and functions in response to abiotic stresses of the RBG gene family remain unclear in *Brassica oleracea*. In this study, a total of 19 *BoiRBG* genes were identified through genome-wide analysis in broccoli. The characteristics of *BoiRBG* sequences and their evolution were examined. An analysis of synteny indicated that the expansion of the *BoiRBG* gene family was primarily driven by whole-genome duplication and tandem duplication events. The *BoiRBG* expression patterns revealed that these genes are involved in reaction to diverse abiotic stress conditions (i.e., simulated drought, salinity, heat, cold, and abscisic acid) and different organs. In the present research, the up-regulation of *BoiRBGA13* expression was observed when subjected to both NaCl-induced and cold stress conditions in broccoli. Moreover, the overexpression of *BoiRBGA13* resulted in a noteworthy reduction in taproot lengths under NaCl stress, as well as the inhibition of seed germination under cold stress in broccoli, indicating that RBGs play different roles under various stresses. This study provides insights into the evolution and functions of *BoiRBG* genes in *Brassica oleracea* and other Brassicaceae family plants.

## 1. Introduction

Glycine-rich proteins (GRPs), which were first identified and characterized in *Petunia* and *Cucurbita* species [1], are a group of proteins consisting of glycine residues with (Gly)n-X repetitions [2]. GRPs play a key role in regulating gene expression at the transcriptional level (including transcription factors and histone modifications) and post-transcriptional level (encompassing pre-mRNA splicing, capping, and polyadenylation as well as mRNA transport and translation) during plant developmental processes, hormone responses, and responses to various environmental stresses [3,4,5,6]. More specifically, GRPs have been grouped into five classes (I, II, III, IV, and V) based on additional conserved motifs and the arrangement of glycine repeats [3,7]. The current study examined Class IV representing RNA-binding glycine-rich proteins (RBGs), which include an RNA-recognition motif (RRM) or a cold-shock domain (CSD) in addition to glycine-rich C-terminals, with or without a zinc-finger domain [7]. RBGs have also been subdivided into the following four subclasses based on their domain arrangement: Class IVa (one RRM motif in addition to the glycine-rich domain), Class IVb (one RRM and a CCHC zinc finger in addition to the glycine-rich domain), Class IVc (a CSD and two or more zinc fingers in addition to the glycine-rich domain), and Class IVd (two RRMs, along with the glycine-rich domain) [7]. Moreover, the RRM consists of the following two highly conserved motifs: ribonucleoprotein (RNP)-1, characterized by eight amino acid residues [(K/R)G(F/Y)(G/A)FVX(F/Y)], and RNP-2, featuring six amino acids [(L/I)(F/Y)(V/I)(G/K)(G/N)L] [8]. The RRM and CSD play a key role related to RNA-binding activity, with the glycine-rich domain contributing to RNA binding by inducing discrete secondary structures and enhancing the interaction of RBGs with other proteins and macromolecules [9]. Furthermore, CCHC zinc fingers influence RNA binding by embedding in the glycine-rich domain [10]. There is increasing evidence suggesting that RBGs help mediate responses to various abiotic stresses, with the expression of the corresponding genes up-regulated or down-regulated after exposures to cold, heat, simulated drought, abscisic acid (ABA), and high salinity [10,11,12].

An increasing number of RBG genes have been isolated and identified in diverse species, including *Arabidopsis thaliana* (Arabidopsis) [13,14,15], *Oryza sativa* [13,14,16], *Zea mays* [14,17], *Brassica rapa* [14,18], *Ipomoea trifida* [19], *Gossypium arboreum* and *Gossypium raimondii* [20], *Vitis vinifera* [21]. In addition to being the first to reveal the Class IVd *GRP* genes in Arabidopsis and rice, Krishnamurthy screened the whole genomes of Arabidopsis and rice and uncovered more RBG genes (IVa = 7; IVb = 3; IVc = 4; and IVd = 5 in Arabidopsis and IVa = 6; IVb = 2; IVc = 2; and IVd = 4 in rice) than an earlier study (IVa = 8; IVb = 3; and IVc = 2 in Arabidopsis and IVa = 6; IVb = 3; and IVc = 2 in rice) [13,14]. By removing distantly related and unrelated proteins, members of the *RBG* gene class were more precisely identified in maize [14]. In these previous studies, RBG genes were associated with different functions under various stress conditions because of the presence of diverse domains. For example, at least two distinct secondary structures can form to facilitate the RNA-binding activities of glycine-rich domains [9]. Both *AtRBG2* and *AtRBG7* contribute to cold tolerance by encoding RNA chaperones, with the overexpression of *AtRBG2* promoting freezing tolerance in Arabidopsis [22,23]. Additionally, *AtRBG7* expression is regulated by plant hormones, such as ABA, with implications for responses to the osmotic stress caused by drought or high salinity. Specifically, AtRBG7 helps mitigate the adverse effects of water deficit stress by regulating the stomatal aperture [24]. Moreover, the previous studies of *AtGRP7* indicate that it is involved in the process of flowering transition in an autonomous pathway; *AtGRP7* was also previously demonstrated to primarily regulate flowering time by influencing the MADS-box repressor *FLOWERING LOCUS C* (*FLC*) [25,26].

*Brassica oleracea* (2n = 2x = 18) (CC) belongs to the Brassicaceae family and is one of three diploid Brassica species in the triangle of U, which includes diploids *Brassica rapa* (AA) and *Brassica nigra* (BB). Additionally, it shares a genetic relationship with radish (*Raphanus sativus*) (RR). Moreover, *Brassica oleracea* encompasses various subspecies, including broccoli, cabbages, kale, and cauliflower, each contributing to its diverse botanical profile. The number of genomic studies regarding Cruciferae species, including broccoli, has recently increased [27,28,29,30,31], with the generated data helping to characterize gene families. However, a comprehensive exploration of the *B. oleracea* RBG genes has not been conducted.

The current study involved a thorough analysis of the *RBG* gene families within *B. oleracea* and other Brassicaceae family species (*R. sativus* and *B. nigra*) based on genomic data [26,27,28,29,30]. Moreover, the *B. oleracea RBG* gene family was characterized in terms of chromosomal distributions, phylogenetic relationships, exon–intron organization, encoded conserved motifs, tandem duplications and synteny, as well as their expression profiles across different tissues and under the influence of abiotic stressors. More specifically, the *RBG* superfamily was named based on the systematic nomenclature formulated by Krishnamurthy, and Subclass IVc members were excluded from the RBG class [14]. The findings of our study may provide the foundation for future research aimed at a deeper understanding of the RBG genes in *B. oleracea* and its closely related species.

## 2. Results

### 2.1. Genome-Wide Identification and Characterization of the RBG Gene Family Members

Using all identified *AtRBG* genes as reference sequences, broccoli, kale, head cabbage, radish, and black mustard RBG genes were identified by screening for homologs and confirming the presence of conserved domains. Phylogenetic trees constructed with the members of all four subclasses revealed that IVa, IVb, and IVd members were clustered together with IVc members to form a distinct clade (Figure S1). Considering IVc subclass members were classified with the CSD superfamily in previous studies [32,33], we removed IVc members from the *RBG* family, similar to a previous study [14].

On the basis of sequence homologies and conserved domains, 19 *BoiRBG* genes were identified in *B. oleracea* HDEM, with 14, 2, and 3 genes in Subclasses IVa, IVb, and IVd, respectively (Table S1). Moreover, 20 (IVa = 11; IVb = 3; IVd = 6), 18 (IVa = 12; IVb = 2; IVd = 4), 16 (IVa = 10; IVb = 2; IVd = 4) and 15 (IVa = 9; IVb = 3; IVd = 3) proteins were designated as RBGs of *B. oleracea* TO1000DH, *B. oleracea* 02–12, *R. sativus* and *B. nigra*, respectively. The glycine residue content of all RBGs exceeded 20%. 

**Figure 1:**
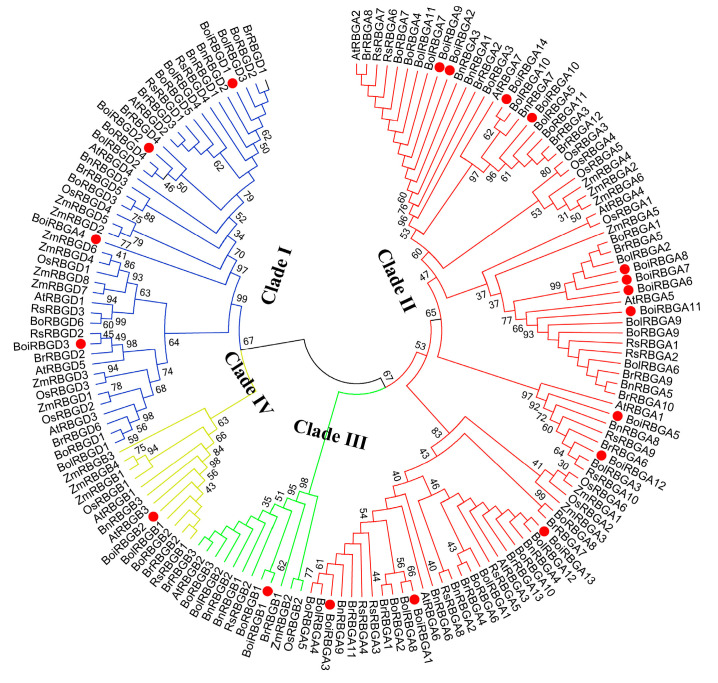
Phylogenetic relationships among RBGs from *B. oleracea* var. *broccoli* and other species. The proteins in the Clade I are presented in blue. The proteins in Clade II are presented in red. The Clade III and Clade IV proteins are presented in green and yellow, respectively. The identified *B. oleracea* var. *broccoli* RBGs are indicated with a red circle. Species abbreviations are defined in Table S2.

To clarify the evolutionary relationships of these RBG family members, we constructed a neighbor-joining phylogenetic tree comprising 22 RBG proteins from Chinese cabbage, 18 from maize, 12 from rice, and 15 from Arabidopsis in addition to 88 RBG proteins identified in the current study through a multiple sequence alignment (Figure 1 and Table S2). The phylogenetic tree branch of Arabidopsis was consistent with the previous research [14]. The phylogenetic tree indicated that 155 RBGs were clustered into four clades (Figure 1). Specifically, Clade I contained all RBGDs from nine species, whereas Clade II included all RBGAs from nine species, with the exception of BoiRBGA4. The RBGBs were distributed in Clades III and IV.

The physical and chemical properties of all 88 RBG genes and the encoded proteins were analyzed (Table S1). The isoelectric point of RsRBGD4 was not calculated because of the presence of several consecutive undefined amino acids. The 19 BoiRBG proteins comprised 104–545 amino acids, with predicted molecular weights and isoelectric points of 10.62 to 56.53 kDa and 4.61 to 10.06, respectively. Of the 19 BoiRBGs, BoiRBGA6 was identified as the shortest, with the lowest molecular weight and isoelectric point. The differences among the proteins were mostly due to variations in the nonconserved amino acid regions. 

### 2.2. Chromosome Location and Sequence Similarity Analysis of BoiRBG Genes

**Figure 2:**
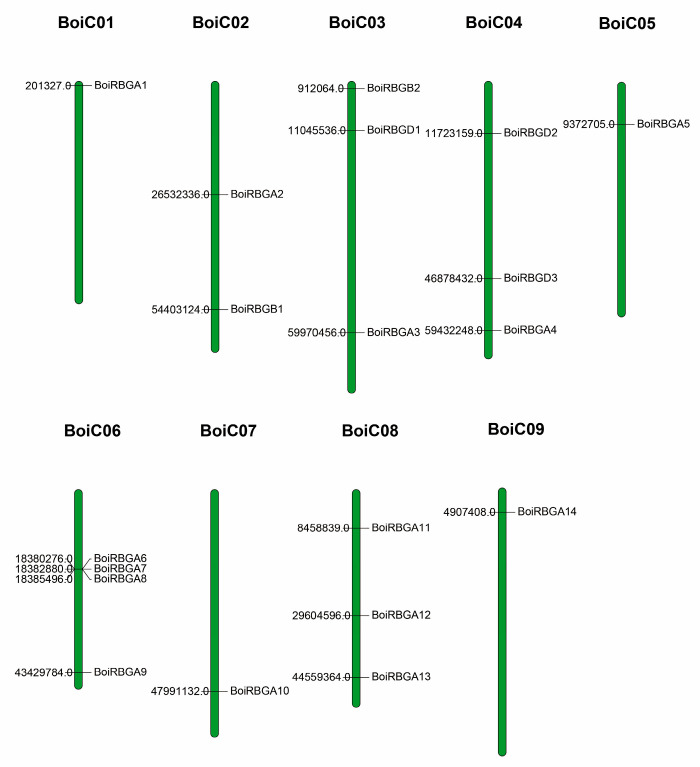
Distribution of BoiRBG genes on *B. oleracea* var. broccoli chromosomes. The line on the green bars indicates the location of RBG genes on chromosomes. The values on the left refer to the chromosomal physical distances.

**Figure 3:**
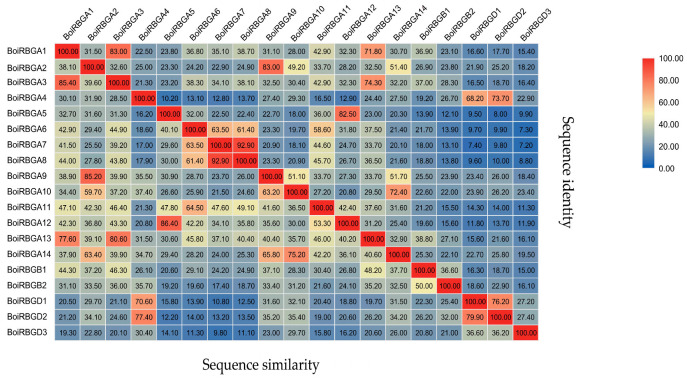
*Brassica oleracea* var. *broccoli* RBG amino acid sequence identities and similarities (%).

The current research primarily focused on the RBGs of broccoli, and the sequence analysis of BoiRBGs was carried out. *BoiRBG*s were distributed on all nine chromosomes (BoiC 01–BoiC 09). Additionally, BoiC 06 contained four RBG genes, which was the most of any of the HDEM chromosomes, followed by BoiC 03, BoiC 04, and BoiC 08 with three RBG genes each (Figure 2). A comparison of the *BoiRBG* sequences revealed a nucleotide sequence identity of 7.2–83%, with a 9.8–86.4% sequence identity at the amino acid level (Figure 3). Moreover, BoiRBGA4 was also classified into the IVa group with only one RRM domain, even though it shows high sequence similarity and sequence identity with BoiRBGD1 and BoiRBGD2 (Figure 3 and Table S1).

### 2.3. Conserved Domain Compositions and Structural Analysis of BoiRBG Genes

**Figure 4:**
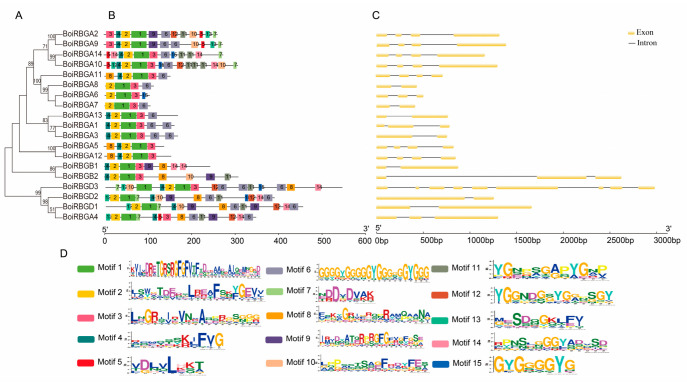
Phylogenetic, motif structure, and gene structure analyses of BoiRBGs. (**A**) Phylogenetic tree comprising BoiRBG proteins. (**B**) Schematic representation of the predicted conserved BoiRBG motifs. (**C**) Exon–intron structures in *BoiRBG* genes. (**D**) Fifteen different motifs are represented by colored boxes.

To further investigate the diversity and similarity of the BoiRBG motif compositions, another phylogenetic tree of BoiRBG protein sequences was constructed (Figure 4A). The data indicated that BoiRBGA1–12 (except for BoiRBGA4) clustered together. Additionally, BoiRBGB1–2 were grouped together, as were BoiRBGD1–3 and BoiRBGA4. Thus, BoiRBGA4 may be closely related to IVd members.

**Figure 5:**
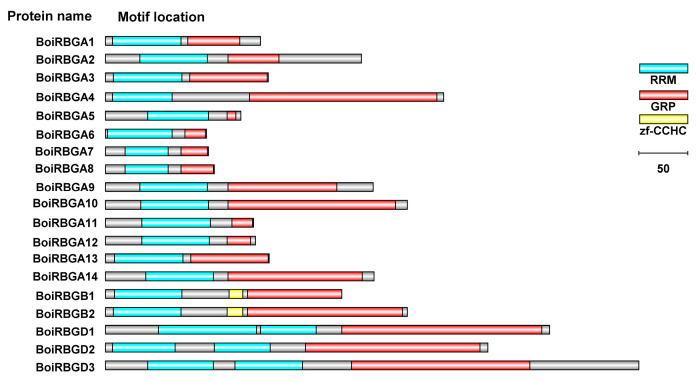
Motif compositions of BoiRBGs.

To analyze the confirmed motifs, multiple BoiRBG sequences were aligned with MultAlin, and the motif compositions were examined with IBS1.0 based on the screening of the Pfam database. The results confirmed that all 19 BoiRGBs were rich in glycine residues at the C-terminal and contained two consensus RNPs with or without a zinc-finger domain, which was consistent with the earlier results (Figure 5 and Figure S2) [7]. 

To further clarify the diversity and similarity of BoiRBGs in *B. oleracea* HDEM, the potential motifs were predicted with MEME (Figure 4B). The results proved that most of the RBGs in the same subclass share similar motifs, implying they are functionally similar.

To explore the structural variations among BoiRBGs, the exon–intron organization of *BoiRBG* genes was analyzed and plotted. These genes included 1–8 introns, with most comprising 1–3 introns, whereas *BoiRBGD3* consisted of eight introns. The position and length of the introns differed substantially (Figure 4C). A more thorough inspection proved that the closely related genes had more similar exon–intron structures than the distantly related genes, suggesting that they may have a relatively close evolutionary relationship. Additionally, the BoiRBGs differed regarding the predicted subcellular localization, with nine in the nucleus, six in the cytoplasm, three in the chloroplast, and one in the mitochondrion (Table S1).

### 2.4. Synteny Tandem Duplications of RBG Genes

Collinear orthologs have an important relationship with gene evolution, and tandem duplications influence genome complexity and evolution [34]. The potential duplications and the evolution of RBG genes may be indicated by the distribution of paralogs. An analysis conducted using the SynOrths program revealed that these syntenic relationships resulted from events such as whole-genome triplication (WGT) or segmental duplications. The syntenic and tandem relationships of RBG genes between Arabidopsis and kale, head cabbage, radish, and black mustard were investigated to trace the evolutionary history of RBG genes (Tables S3 and S4 and Figure S3). Both *AtRBGB1* and *AtRBGA4* lacked a syntenic gene in the analyzed species. Additionally, *Bo7g019730*, *Bo8g023090*, *Bo03121s010*, and *Bo2g075560* in kale, as well as *Bol003395*, *Bol006195*, and *Bol022773* in head cabbage, lacked syntenic genes in Arabidopsis. Moreover, *AtRBGD1*, *AtRBGD3*, and *AtRBGD4* had no syntenic genes in broccoli, whereas *AtRBGD3*, *AtRBGD4*, and *AtRBGA7* had no syntenic genes in radish. Similarly, *AtRBGA1*, *AtRBGA5*, and *AtRBGD5* lacked syntenic genes in kale; *AtRBGD1*, *AtRBGD4*, and *AtRBGD5* had no syntenic genes in head cabbage; and *AtRBGD1*, *AtRBGD3* and *AtRBGD5* had no syntenic genes in black mustard. Interestingly, of the 15 *AtRBG* genes, *AtRBGA5* had relatively more syntenic copies in broccoli. Furthermore, the *AtRBGA5* syntenic gene *BoiRBGA6* was tandemly duplicated (Table S4). These results implied that WGT and tandem duplication events contributed to the expansion of the RBG gene family in broccoli, whereas WGT was more important during the evolution of RBG genes in kale, head cabbage, radish, and black mustard.

### 2.5. Expression Profiles of BoiRBG Genes in Various B. oleracea HDEM Organs

**Figure 6:**
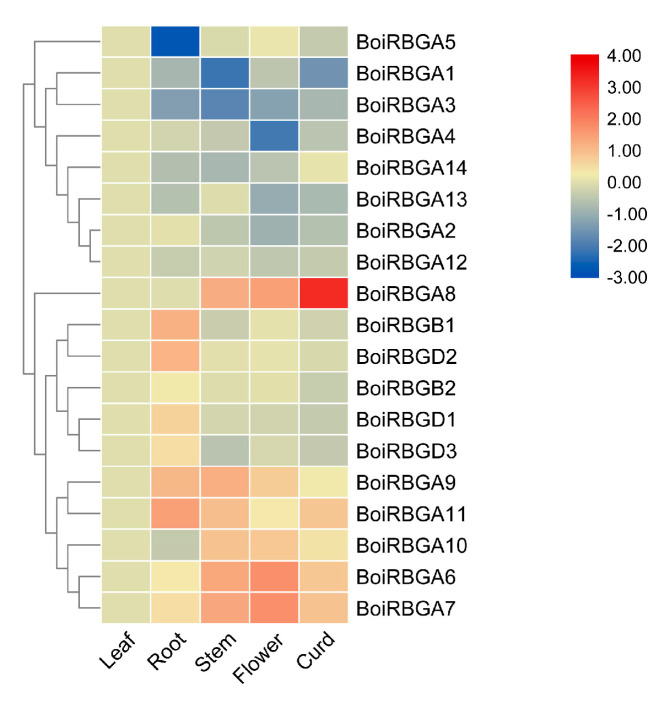
Heat map of *B. oleracea* var. *broccoli BoiRBG* gene expression profiles in various tissues. The log2-transformed expression values (2^−ΔΔCt^) were used to visualize the relative expression levels of *BoiRBGs* across various broccoli tissues. Different colors corresponded to distinct relative expression levels in the color scale. Leaf tissue was considered as control samples, resulting in a value of (0).

To clarify the biological functions of the 19 *BoiRBG* genes, their expression patterns were analyzed in various organs (flowers, leaves, stems, curds, and roots) via qRT-PCR (Table S5). The generated heat map illustrated that the expression levels of the 19 *BoiRBG* genes varied among the examined tissues (Figure 6). Specifically, *BoiRBGA5* was expressed at low levels in the roots, while the expression level of *BoiRBGB1* and *BoiRBGD2* in the root was relatively high. Using hierarchical cluster analysis, we grouped the *BoiRBG* genes into two distinct categories according to their expression profiles. However, these groups were inconsistent with the subgroup categorizations of IV RBG genes. For the tandemly duplicated genes, *BoiRBGA6* and *BoiRBGA7* were similarly expressed and were clustered together, whereas *BoiRBGA8* was differentially expressed, with high curd expression levels, implying it has a diverse role (Figure 6).

### 2.6. Analysis of BoiRBG Expression in Response to Different Abiotic Stresses in Broccoli

**Figure 7:**
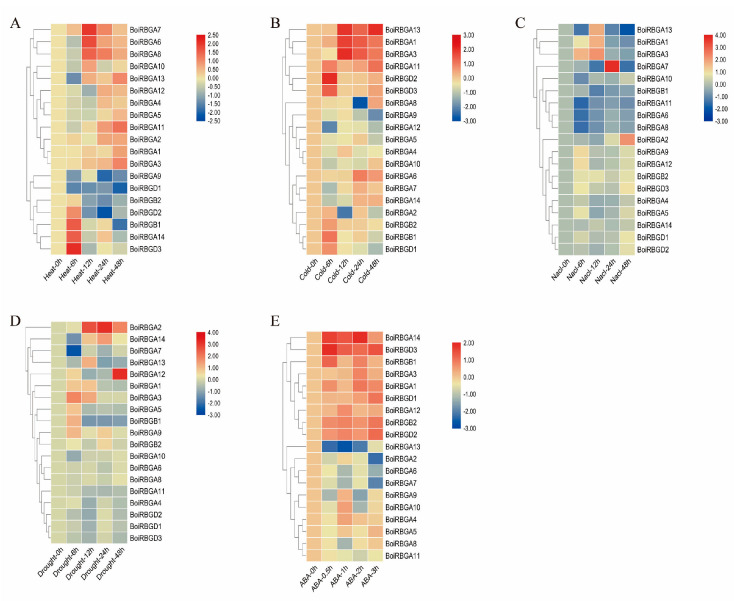
Heat maps of *BoiRBG* expression levels in leaves under various abiotic stress conditions. (**A**) Heat stress; (**B**) Cold stress; (**C**) Nacl stress; (**D**) Drought stress; (**E**) ABA stress. The log2-transformed expression values (2^−ΔΔCt^) were used to visualize the relative expression levels of *BoiRBGs* under various abiotic stress conditions. Different colors corresponded to distinct relative expression levels in the color scale. 0 h issue was considered as control samples, resulting in having a value of (0).

To examine the responsiveness of *BoiRBG* genes to abiotic stresses, the expression of all 19 *BoiRBG* genes following exposures to different stresses, including high and low temperatures, salinity, ABA, and simulated drought, were analyzed via qRT-PCR. The temporal expression patterns of these genes are presented as heat maps (Figure 7). Heat stress up-regulated the expression levels of the tandemly duplicated genes (*BoiRBGA6*, *BoiRBGA7*, and *BoiRBGA8*), which were highly expressed at 12 h. In contrast, the expression of *BoiRBGD1* and *BoiRBGA9* tended to decrease, reaching the lowest level at 48 h (Figure 7A). Regarding the low-temperature treatment, *BoiRBGA1*, *BoiRBGA3*, and *BoiRBGA13* expression levels were slightly down-regulated at 6 h and then up-regulated, peaking at 12 h before decreasing, whereas *BoiRBGA11*, *BoiRBGD2*, and *BoiRBGD3* expression was slightly up-regulated at 6 h and then down-regulated (Figure 7B). Under salt stress conditions, all *BoiRBGD* expression levels were slightly up-regulated. Additionally, *BoiRBGA1*, *BoiRBGA3*, and *BoiRBGA13* expression levels were up-regulated, peaking at 12 h, after which they were down-regulated. The expression of *BoiRBGA7* increased considerably at 24 h but exhibited only slight changes at the other time points (Figure 7C). Drought stress did not substantially change gene expression levels, except for *BoiRBGA1*, *BoiRBGA2*, *BoiRBGA3*, *BoiRBGA7*, *BoiRBGA12*, *BoiRBGA13*, and *BoiRBGA14.* Specifically, *BoiRBGA2* expression was considerably up-regulated at 12, 24, and 48 h. The *BoiRBGA1*, *BoiRBGA3*, and *BoiRBGA13* expression levels were initially up-regulated and then down-regulated, whereas the *BoiRBGA7* expression levels exhibited the opposite pattern (Figure 7D). The *BoiRBGA14* and *BoiRBGD3* expression levels were considerably up-regulated by the ABA treatment, whereas ABA had the opposite effect on *BoiRBGA13* expression (Figure 7E). The varied expression of these *BoiRBG* genes in response to different stresses indicates potential differences in their roles; however, further confirmation is necessary.

### 2.7. BoiRBGA13 Was Involved in the Regulation of Seed Germination and Seedling Growth under NaCl and Cold Stress in Broccoli

The above studies showed that the expression of the *BoiRBG* gene responds to various stresses, among which the expression of *BoiRBGA13* significantly changed under NaCl and cold conditions. To further elucidate the function of *BoiRBGs*, we used BoiRBGA13 transgenic broccoli as an example to investigate the impact of NaCl and cold stress on seed germination and seedling growth in three independent T3 transgenic lines.

**Figure 8:**
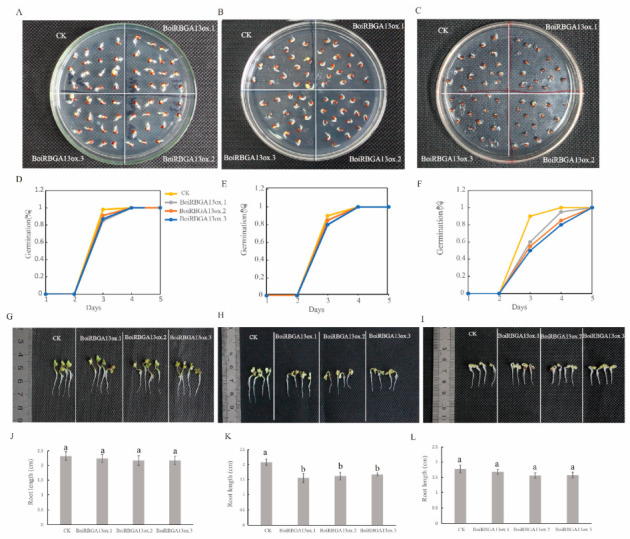
*BoiRBGA13* involved in NaCl and cold stress. (**A**–**C**) the photo of the seed germination of *BoiRBGA13*-overexpressed broccoli under normal, NaCl (200 mM) and cold (13 °C) treatments. (**D**–**F**) Statistic results of the seed germination rate of *BoiRBGA13*-overexpressed broccoli under normal, NaCl and cold treatments. (**G**–**I**) the photo of the seedling of *BoiRBGA13*-overexpressed broccoli under normal, NaCl (200 mM) and cold (13 °C) treatments. (**J**–**L**) Taproot length was measured on the fourth day after NaCl and cold treatment. Small letter(s) above the bars indicate significant differences (α = 0.05, LSD) among the treatments.

The results indicated that there were no significant differences in germination potentials and seedling phenotypes among the transgenic lines (BoiRBGA13ox.1, BoiRBGA13ox.2, BoiRBGA13ox.3) and the control wild-type (CK) under normal environmental conditions (Figure 8A,D,G,J). The germination potentials of *BoiRBGA13* transgenic lines exposed to 200 mmol·L^−1^NaCl treatment were not significantly different from that of the control (Figure 8B,E), but the seedling lengths of all the transformed plants were shorter, and taproot lengths were significantly shorter (Figure 8H,K). More interestingly, all the transformed plants showed an exaggerated apical hook phenotype after four days of growth under NaCl stress conditions, in contrast to the control group, consistent with the triple response of ethylene (Figure 8H). Under cold stress, the germination potentials of *BoiRBGA13* transgenic lines were observed to be 60%, 55% and 50%, respectively, demonstrating a significant decrease compared to the control (90%) (Figure 8C,F). Nevertheless, there was no statistically significant difference in the taproot length between these transgenic lines and the control group (Figure 8I,L), but the exaggerated apical hook phenotype was kept in the transgenic seedlings. Hence, the results suggest that *BoiRBGA13* plays different roles in seed germination and seedling growth under different stresses in broccoli.

## 3. Discussion

The RBG genes form a superfamily with important regulatory roles affecting gene expression across various tiers (e.g., by altering processes such as alternative splicing, export, translation, and RNA degradation) [2,10]. Because of their key roles and the increasing availability of genomic data, researchers have identified multiple RBG genes in many species, such as Arabidopsis, rice, maize, wheat, cotton, sweet potato, and tobacco [14,17,19,20,35,36,37]. In the current research, we identified RBG genes in *B. oleracea*, *R. sativus* and *B. nigra*. The phylogenetic relationships, gene and protein structures, and glycine contents were analyzed based on the known Arabidopsis RBG genes. We also conducted analyses of gene and protein structures, identified conserved motifs, and investigated variations in expression patterns across different tissues and in response to varying conditions. Our findings suggest the *BoiRBG* genes are moderately similar regarding their overall sequences and functions but vary somewhat regarding their tissue-specific roles and responses to diverse stresses. Thus, the data presented herein may provide insights into the *BoiRBG* family and help researchers functionally characterize these genes.

The RBG (equivalent to IV GRPs) are distinguished from other GRPs by their nucleic acid-binding domains, with Subclasses IVa, IVb, IVc, and IVd differing in terms of the domain arrangement [7,38]. Although the members of all four subclasses can bind RNA, this activity is mediated by RRM domains in IVa, IVb, and IVd, whereas in IVc, it is due to the CSD, which can also bind DNA by interacting with one or two CCHC zinc fingers [3,10]. Accordingly, we excluded Subclass IVc from the RBG family, similar to previous studies [14,19]. Furthermore, glycine-rich C-terminal domains in RBGs might contribute to the RNA-binding activity of the RRM and facilitate protein–protein interactions by giving rise to discrete secondary structures such as glycine loops and β-sheets [10]. All of the RBGs identified in the present study contained one or two RRM domains with a glycine-rich C-terminal (20–70% of residues), enabling the RNA-binding activity and playing key roles in the regulation of mRNAs, with high expression levels induced by diverse abiotic stresses. Moreover, the differences in the subcellular localization of the RBGs identified in this study may be related to the functional diversity of these proteins in plants. Analyses of motif locations and exon–intron structures revealed similarities in gene structures, which contribute to functional similarities. 

On the basis of the phylogenetic analysis, all RBGs were classified into four clades, with some RBGB members more closely related to RBGAs, whereas others were more closely related to RBGDs, implying that RBGBs may have derived from different ancestors. Furthermore, two phylogenetic trees confirmed the relatively close relationship between some RBGBs and RBGAs, which was consistent with the results of earlier investigations that indicated RBGAs and some RBGBs share a common ancestor [14,19]. These findings confirmed the validity of our predictions. Moreover, *BoiRBGD2* and *BoiRBGA4* had comparatively high sequence similarity and shared the same clade in phylogenetic analysis, which suggests that they may have the same function with different domain arrangement. The exon–intron structures and motif arrangements suggest the *BoiRBG* genes are functionally similar [39].

Gene duplication events are important for expanding some gene families and diversifying gene functions [40]. Approximately 14–20 million years ago, the common ancestor of *Brassica* crop species and *R. sativus* underwent a WGT event after diverging from Arabidopsis [41,42]. In our study, we identified 19, 20, 18, 16, and 15 members of the RBG gene superfamily in broccoli, kale, head cabbage, radish, and black mustard, respectively. This count is notably lower than the triplicated number of RBG gene family members found in *Arabidopsis thaliana* (45), indicating that more than half of the RBG genes were lost in the aftermath of WGT events in these genomes. The varying numbers of retained syntenic genes in the analyzed species may be attributed to gene loss events, which could have played a role in maintaining metabolic balance after genomic duplications [43]. No syntenic copies of *AtRBGA4* and *AtRBGB1* were detected in the examined species, which is similar to the findings from a prior study of Chinese cabbage by Krishnamurthy, indicating that the loss of *AtRBGA4* and *AtRBGB1* occurred in the common ancestor of these six species [14].

*AtRBGs* display tissue- and organ-specific expression patterns during Arabidopsis development, exhibiting the highest expression levels in shoot apex, vegetative rosette, seeds, and flowers [6]. This observation partially aligned with the expression patterns of *BoiRBGs* in different broccoli tissues. *BoiRBGs* exhibit robust RNA recognition and binding capabilities, suggesting its potential involvement in the molecular mechanisms governing plant growth and development through splicing activation or transcriptional regulation [6]. Notably, *BoiRBGA8* was highly expressed in the curd and expressed at lower levels in the stems and flowers. Thus, future studies should investigate whether this gene is involved in curd development. Otherwise, *BoiRBGA6/7/8* originated through tandem duplication, a significant mechanism for the emergence of young genes [44]. Young genes typically exhibit biased expression in male reproductive tissues. Interestingly, these three genes also displayed biased expression in flowers and curd (Figure 6), suggesting that they have evolved critical male reproductive functions and could potentially be considered evolutionary young genes as well [45]. It is conceivable that the deletion of these three tandem duplicates may lead to male sterility phenotypes, offering novel insights into the functionality of the RBG gene family.

The expression of *RBGs* can be induced by various stresses and change with different species, indicating that they play different roles in terms of stress resistance. In Arabidopsis, *AtGRP2* and *AtGRP7* play important roles in plant adaptations to cold, with expression levels that are up-regulated by low temperatures [5,13,22,23,46]. Under conditions of drought and salt stresses, there was an increase in the expression of *AtGRP1*, while the expression of *AtGRP4* and *AtGRP7* showed a gradual decrease, and the expression of *AtGRP5* and *AtGRP6* remained unchanged [46]. Moreover, *NtGRP1* had low expression under drought, salt, and high and low temperature stress but high expression at the early stage of waterlogging stress in tobacco [47]. Out of the four glycine-rich RNA-binding protein genes (*OsRBP1* to *OsRBP4)* in rice, only *OsRBP4* exhibited high expression levels under conditions of high temperature [16]. In the present research, a total of 19 *BoiRBGs* were found to have differential expression in response to high and low-temperature stress, drought and salt stresses. These genes displayed distinct expression profiles under varying conditions of stress tolerance, indicating their diverse roles in the process of plant adaptation to adversity. The expression of *BoiRBGA13* was up-regulated due to drought stress, which is consistent with the reported effects of *AtGRP7* on stomatal regulation in Arabidopsis [24]. However, the expression of *BoiRBGA2* was also induced by drought conditions, which has not been reported in previous studies, and the underlying regulatory mechanism is unclear. Therefore, future evaluations of the drought stress resistance of *B. oleracea* should consider the role of *BoiRBGA2*. The expression levels of *BoiRBGA13* and *BoiRBGA1*, as well as *BoiRBGA3*, were considerably induced by cold and NaCl stress and had the same expression profiles in our research, implying these genes may have relative functions related to cold and NaCl stress in *B. oleracea*.

Distinct expression patterns of the same *BoiRBG* gene under different stress conditions indicate that they perform varied functions in response to different stresses. In Chinese cabbage, two *BrGRP* genes, *w546 (Bra028063)* and *w1409 (Bra014000)*, were notably up-regulated under salt stress, and *w546* can decrease seed germination potential under salt stress without influencing it under mannitol stress; however, *w1409* exhibits a completely opposite function [18]. Furthermore, *BoiRBGA13* had been characterized as a regulator suppressing taproot length in response to NaCl exposure and diminishing the seed germination potential under cold stress in transgenic broccoli plants, indicating that *BoiRBGA13* had different molecular responses to various stresses. In Arabidopsis, the expression of *AtGRP7* and *AtGRP8* can be induced by cold stress [3]. Interestingly, the corresponding homologous genes in broccoli, *BoiRBGA13* (homologous to *AtGRP7*) and *BoiRBGA1* and *BoiRBGA3* (homologous to *AtGRP8*), displayed similar expression profiles under both cold and NaCl treatments. This may be attributed to specific motifs in their promoters or protein domains, providing novel insights into the functional analysis of *BoiRBGA13*, *BoiRBGA1*, and *BoiRBGA3*.

The development of the apical hook in seedlings is regulated by various hormones (such as Gibberellins, Ethylene, and Jasmonic acid) and environmental signals [48,49,50]. Interestingly, the transgenic seedlings displayed a pronounced apical hook phenotype under low-temperature or salt stress, which indicates that *BoiRBGA13* may exert a significant influence on seed germination, especially when seedling growth induces stress signals and hormone signals in broccoli.

## 4. Materials and Methods

### 4.1. Sequence Acquisition and Genome-Wide Identification of RBG Genes in B. oleracea

Whole-genome sequences of three *B. oleracea* subspecies were selected. The complete genome sequences of *B. oleracea* ssp. *botrytis broccoli* HDEM were obtained from the Genoscope database (http://www.genoscope.cns.fr/ (accessed on 11 March 2023) [31]. Additionally, *B. oleracea* ssp. *capitata* line 02–12, *R. sativus* XYB-36-2 and *B. nigra* YZ12151 genome resources were downloaded from the BRAD database (http://brassicadb.org/brad/ (accessed on 11 March 2023) [27,29,30]. The whole-genome sequences of *B. oleracea* kale-like type TO1000DH were downloaded from the EnsemblPlants website (http://plants.ensembl.org/ (accessed on 11 March 2023) [28]. Considering that the *RBG* family members in Arabidopsis have been identified in many previous studies, the AtRBG amino acid sequences were downloaded from the TAIR database (http://arabidopsis.org/ (accessed on 11 March 2023) and used in conjunction with the synteny analysis and BLASTP 2.9.0 tools to identify homologous RBG genes in the genomes of *B. oleracea*, *R. sativus*, and *B. nigra* [13,14]. Moreover, the initially screened proteins, BoiRBGs (for *B. oleracea* HDEM), BoRBGs (for *B. oleracea* TO1000DH), BolRBGs (for *B. oleracea* 02–12), RsRBGs (for *R. sativus* XYB-36-2) and BnRBGs (for *B. nigra* YZ12151) were further filtered using the Hidden Markov Model profile (version 3.1) matching the Pfam RRM family profiles (PF00076, PF04059, PF08777, PF10378, PF10598, PF13893, and PF14259), with a sequence coverage of Pfam domain models of at least 60% and an E-value cutoff of 1 × 10^−5^ [35,51]. The obtained RBGs with high glycine contents (>20% of the C-terminal residues) were verified with the Simple Modular Architecture Research Tool 9.0 (http://smart.embl-heidelberg.de/ (accessed on 21 March 2023) and InterPro 95.0 (http://www.ebi.ac.uk/interpro/ (accessed on 21 March 2023) [52,53]. Furthermore, the *BoiRBG*, *BoRBG*, *BolRBG*, *RsaRBG*, and *BnRBG* gene sequences (including introns and exons), open reading frames, and encoded amino acid sequences were downloaded from the corresponding genome databases.

The RBGs were named and grouped into four subclasses according to their conserved motif arrangements as previously described, and a multiple sequence alignment was conducted using MultAlin 5.4 (http://multalin.toulouse.inra.fr/multalin/ (accessed on 21 March 2023) [14,54]. Additionally, the physicochemical properties of the RBGs, including the molecular weight, theoretical isoelectric point, and size, were determined with the ExPASy 5.0 online tool (http://expasy.org/tools/ (accessed on 21 March 2023) [55]. Moreover, the reference genome sequences of *B. oleracea* HDEM were considered for subsequent analysis for the highest quality by combining the long-read data. Mapchart 2.32 software was used to visualize the chromosomal locations of RBG genes based on *B. oleracea* HDEM chromosomal information [56].

### 4.2. Sequence Alignment and Phylogenetic Analyses of RBG Genes

We employed the global alignment tool ‘Needle’ from the EMBOSS 6.6.0 software suite to calculate the sequence identities and similarities based on the pairwise alignment of BoiRBG proteins [57]. The amino acid sequences for the RBGs from *B. oleracea*, *R. sativus*, and *B. nigra* were determined in the present study, whereas the sequences of the RBG proteins from the other four species (Arabidopsis, Chinese cabbage, rice and maize) were obtained from a published article [14]. The MUSCLE 3.8.31 program was utilized to align the entire RBG amino acid sequences [58]. We constructed a phylogenetic tree using MEGA software (version 7.0). Specifically, we constructed the phylogenetic tree using the neighbor-joining method based on the Jones–Taylor–Thornton model. We conducted 1000 bootstrap replicates to evaluate the statistical support for each tree node [59]. Additionally, we took into account uniform rates and homogeneous lineages and removed gaps with a site coverage cutoff of 70%. Another phylogenetic tree, specifically for BoiRBG proteins, was also constructed using the same approach.

### 4.3. Detection of Collinear Tandem Duplications and Synteny

To elucidate the evolutionary patterns of RBG genes in *B. oleracea* and its closely related species, we explored both tandem duplications and syntenic relationships among the RBG genes in *B. oleracea*, *R. sativus*, and *B. nigra*. Gene pairs that were separated by 10 or fewer genes and within 200 kb of each other were identified as tandem duplications. We employed the SynOrths 1.0 program to detect syntenic orthologs of RBG genes by considering sequence similarity and the collinearity of flanking genes [60]. The syntenic relationships among the RBG genes in the analyzed species were visualized with the Circos software 0.69 [61].

### 4.4. Conserved Motif, Gene Structure, and Subcellular Localization Analyses of RBG Family Members

We analyzed the *BoiRBG* sequences using the Pfam database (http://pfam.janelia.org/ (accessed on 26 March 2023) to identify the encoded conserved motifs. Schematic diagrams of the motif structures were created with IBS1.0.1 (http://ibs.biocuckoo.org/ (accessed on 26 March 2023) [62]. Additionally, *BoiRBG* structures were examined by comparing the coding and genomic sequences using the Gene Structure Display Server (gsds.cbi.pku.edu.cn/ (accessed on 26 March 2023) [63]. Additionally, details regarding the RBGs were revealed via multiple-sequence alignment with MultAlin 5.4 (http://multalin.toulouse.inra.fr/multalin/ (accessed on 26 March 2023) [54]. The subcellular locations of RBGs were predicted using WoLF PSORT 2017 (http://wolfpsort.org/ (accessed on 26 March 2023) [64].

### 4.5. Plant Materials and Stress Treatments

‘Yanxiu’ broccoli seeds were germinated at 24 °C for 2 days in darkness, after which seedlings were grown in plastic pots containing soil in a controlled-environment greenhouse [16 h light (24 °C)/8 h dark (20 °C)]. To analyze gene expression profiles induced by abiotic treatments, 4-week-old seedlings were divided into six groups, with the control, salt stress, and drought stress groups irrigated with 200 mL water, a 200 mM NaCl solution, and a 300 mM mannitol solution, respectively. For the cold and heat treatments, seedlings were exposed to temperatures of 13 °C and 40 °C, respectively, while being provided with an equal volume of water and exposed to identical light conditions. Leaves samples were collected at 0, 6, 12, 24, and 48 h. Regarding the ABA treatment, 100 mg/L ABA was sprayed on the surface of leaves, which were sampled at 0, 0.5, 1, 2, and 3 h. To examine the tissue-specific expression of RBG genes in broccoli, the root, stem, leaf, and curd were sampled during the middle curd-forming stage, whereas the flower was sampled during the middle floral development stage. Arabidopsis ecotypes Col-0 (Wild-type) and its transgenic line seeds were sown in plastic pots at 22 °C under 16 h light and 18 °C under 8 h dark; the stem apex meristem was sampled.

All collected samples were frozen in liquid nitrogen and stored at −80 °C until analyzed using quantitative real-time polymerase chain reaction (qRT-PCR). Each sample was analyzed with three biological replicates.

### 4.6. BoiRBGA13 Ectopic Expressed in Broccoli

The full-length coding region of *BoiRBGA13* was amplified with specific primers (upstream primer: 5′-TTGGCGCGCCTATGGCGTCCCCTGATGTCGAGTACC-3′; downstream primer: 5′-CCATTTAAATTTACCAACCACCACCACCGCTTC-3′; the underlined section is the enzyme cut site), and the overexpression vector p*35S::BoiRBGA13* was constructed by inserting *BoiRBGA13* under the CaMV35S promoter and NOS terminator of PYBA1302. The broccoli transformation method was consistent with the previous description [65]. We obtained two leaves each from the transgenic plants and the control group. Subsequently, we extracted DNA from these samples using the CTAB (Hexadecyltrimethylammonium bromide) method for PCR analysis. The T3 homologous transgenic seeds were used in our experiments.

### 4.7. NaCl and Cold Stress in Transgenic Broccoli

To investigate the impact of NaCl stress on the germination and seedling growth, T3 homologous transgenic seeds were inoculated on MS medium supplemented with a concentration of 200 mmol·L−1 NaCl. To characterize the affection of cold stress on the germination and seedling growth, T3 homologous transgenic seeds were cultured on MS medium and placed within an incubator maintained at a constant temperature of 13 °C. At least 15 individuals were contained in each treatment and repeated three times. We recorded the number of germinated individuals on a daily basis. Taproot length was measured in MS medium four days after NaCl and cold treatment. The normal environment was as follows: environment temperature 24 °C, light-dark cycle 16/8 h, with humidity approximately 60%.

### 4.8. RNA Isolation and Expression Analysis

Total RNA was extracted from these samples with the RNAprep Pure Kit (Tiangen Biotech, Beijing, China). The RNA integrity was assessed using 1% agarose gel electrophoresis, whereas the RNA purity and concentration were estimated with the NanoDrop 2000 spectrophotometer (ThermoFisher Scientific, Beijing, China). The extracted RNA (1 μg) was used as the template for synthesizing first-strand cDNA with the TIANScript RT Kit (Tiangen Biotech). A qRT-PCR assay was performed with the SYBR Green Kit (Quanshijin, Beijing, China) and the CFX96 Real-time PCR system (Bio-Rad Laboratories, Inc., Hercules, CA, USA). We designed gene-specific primers using the Primer Premier 5.0 program. Additionally, the *UBQ* gene served as an internal reference to normalize gene expression data in broccoli and Arabidopsis samples. The qRT-PCR program was as follows: 95 °C for 5 min; 40 cycles at 95 °C for 10 s and 60 °C for 20 s. Each reaction was completed in triplicate, and the expression levels were calculated with the 2^−ΔΔCt^ method [66].

## 5. Conclusions

In this study, we completed a comprehensive genome-wide identification and functional analysis of the *RBG* gene family in *B. oleracea* and other selected Brassicaceae species. Finally, a total of 19 *BoiRBG* genes were identified in *B. oleracea* HDEM. We also comprehensively characterized the RBG genes by analyzing phylogenetic relationships, chromosomal distributions, gene structures, encoded motif compositions, evolution, and expression profiles across different tissues and in reaction to a range of abiotic stress conditions. The outcomes of this study unveiled both the shared and distinct sequence attributes and functionalities, establishing a basis for prospective explorations into RBG genes within *B. oleracea* and other species.

## Data Availability

Data will be available from the corresponding author upon reasonable request.

## Supplementary Materials

The following supporting information can be downloaded at: https://www.mdpi.com/article/10.3390/plants12213706/s1, Figure S1. Phylogenetic relationships among preliminary identified RBGs from *B. oleracea* var. *broccoli.* The IVc subclass are shown in red; Figure S2. Amino acid sequence alignment of BoiRBGs. The yellow boxes around sequences show conserved motifs, including RRM and glycine-rich domains; Figure S3. Syntenic relationships among *BoiRBG* genes from *B. oleracea* (var. broccoli, acephala, and capitata), *R. sativus*, *B. nigra*, and Arabidopsis were visualized in a Circos plot. The chromosomes of Arabidopsis, *B. oleracea* var. *broccoli*, *B. oleracea* var. *acephala*, *B. oleracea* var. *capitata*, *R. sativus*, and *B. nigra* are shaded in black, blue, green, red, gray, and yellow, respectively; Table S1. Information regarding RBG genes from *B. oleracea* (var. *broccoli*, *acephala*, and *capitata*), *R. sativus*, and *B. nigra*; Table S2. Plant RBG protein sequences used for phylogenetic tree construction; Table S3. Syntenic relationships among the BoiRBGs from Arabidopsis, *B. oleracea* (var. *broccoli*, *acephala*, and *capitata*), *R. sativus*, and *B. nigra*; Table S4. Tandemly duplicated *BoiRBG* genes in *B. oleracea* (var. *broccoli*, *acephala*, and *capitata*), *R. sativus*, and *B. nigra*; Table S5. RT-PCR primers of *BoiRBG* genes.
