# Supplementary material for: Comprehensive Genome-Wide Identification of the RNA-Binding Glycine-Rich Gene Family and Expression Profiling under Abiotic Stress in Brassica oleracea
Source: Plants (Basel). 2023 Oct 27;12(21):3706. doi: 10.3390/plants12213706 (PMC10649936; doi:10.3390/plants12213706)
Supplement: Supplementary file 1 [file plants-12-03706-s001.zip › Figure S3.pdf]

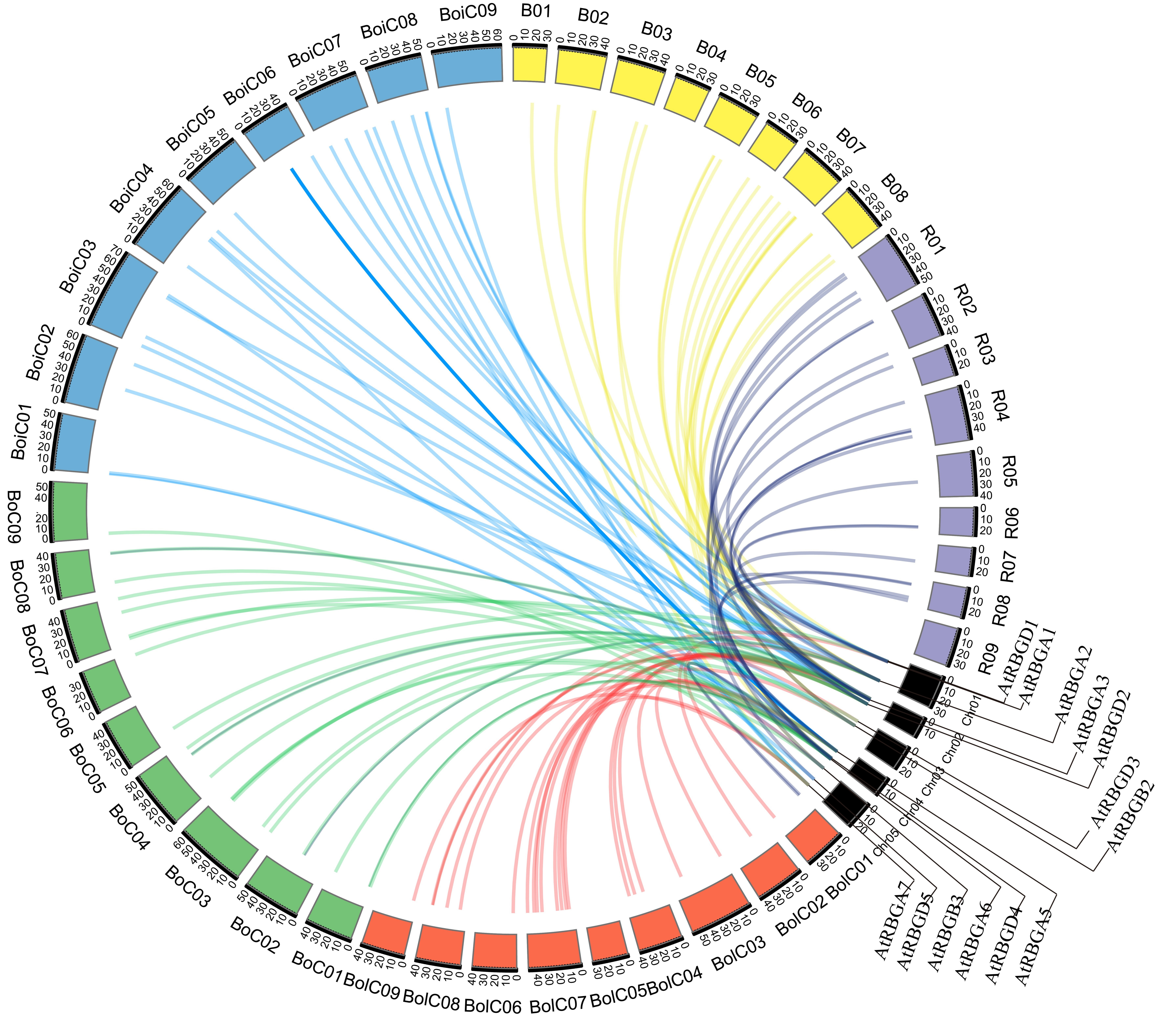

Figure S3. Syntenic relationships among *BoiRBG* genes from *B. oleracea* (var. *broccoli*, *acephala*, and *capitata*), *R. sativus*, *B. nigra,* and Arabidopsis were visualized in a Circos plot. The chromosomes of Arabidopsis*, B. oleracea* var. *broccoli*, *B. oleracea* var. *acephala*, *B. oleracea* var. *capitata*, *R. sativus*, and *B. nigra* are shaded in black, blue, green, red, gray, and yellow, respectively.
