# Supplementary material for: Comprehensive Genome-Wide Identification of the RNA-Binding Glycine-Rich Gene Family and Expression Profiling under Abiotic Stress in Brassica oleracea
Source: Plants (Basel). 2023 Oct 27;12(21):3706. doi: 10.3390/plants12213706 (PMC10649936; doi:10.3390/plants12213706)
Supplement: Supplementary file 1 [file plants-12-03706-s001.zip › Figure S1.pdf]

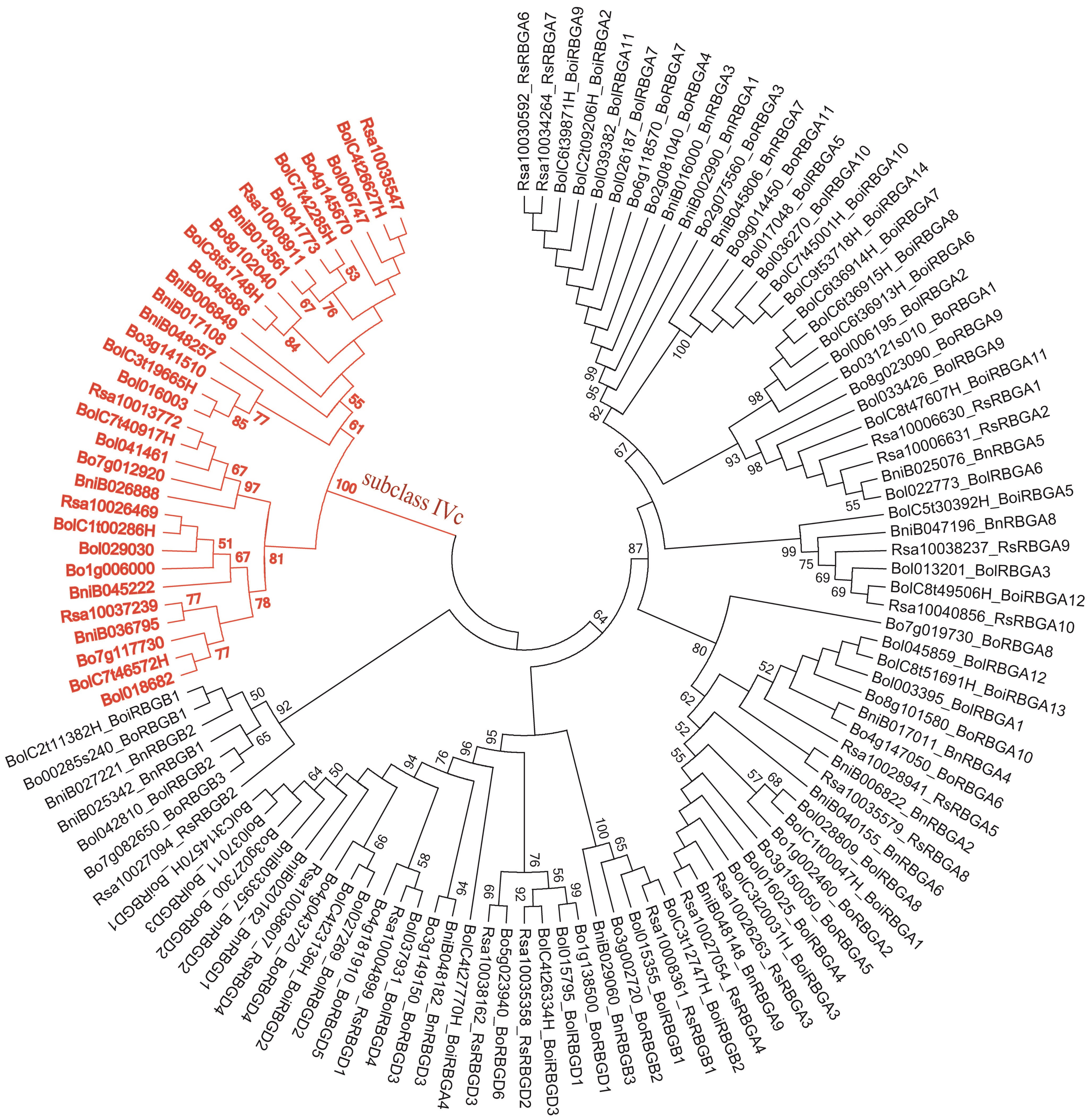

Figure S1. Phylogenetic relationships among preliminary identified RBGs from *B. oleracea* var. *broccoli*. The IVc subclass are shown in red.
