# Supplementary material for: Comprehensive Genome-Wide Identification of the RNA-Binding Glycine-Rich Gene Family and Expression Profiling under Abiotic Stress in Brassica oleracea
Source: Plants (Basel). 2023 Oct 27;12(21):3706. doi: 10.3390/plants12213706 (PMC10649936; doi:10.3390/plants12213706)
Supplement: Supplementary file 1 [file plants-12-03706-s001.zip › Figure S2.pdf]

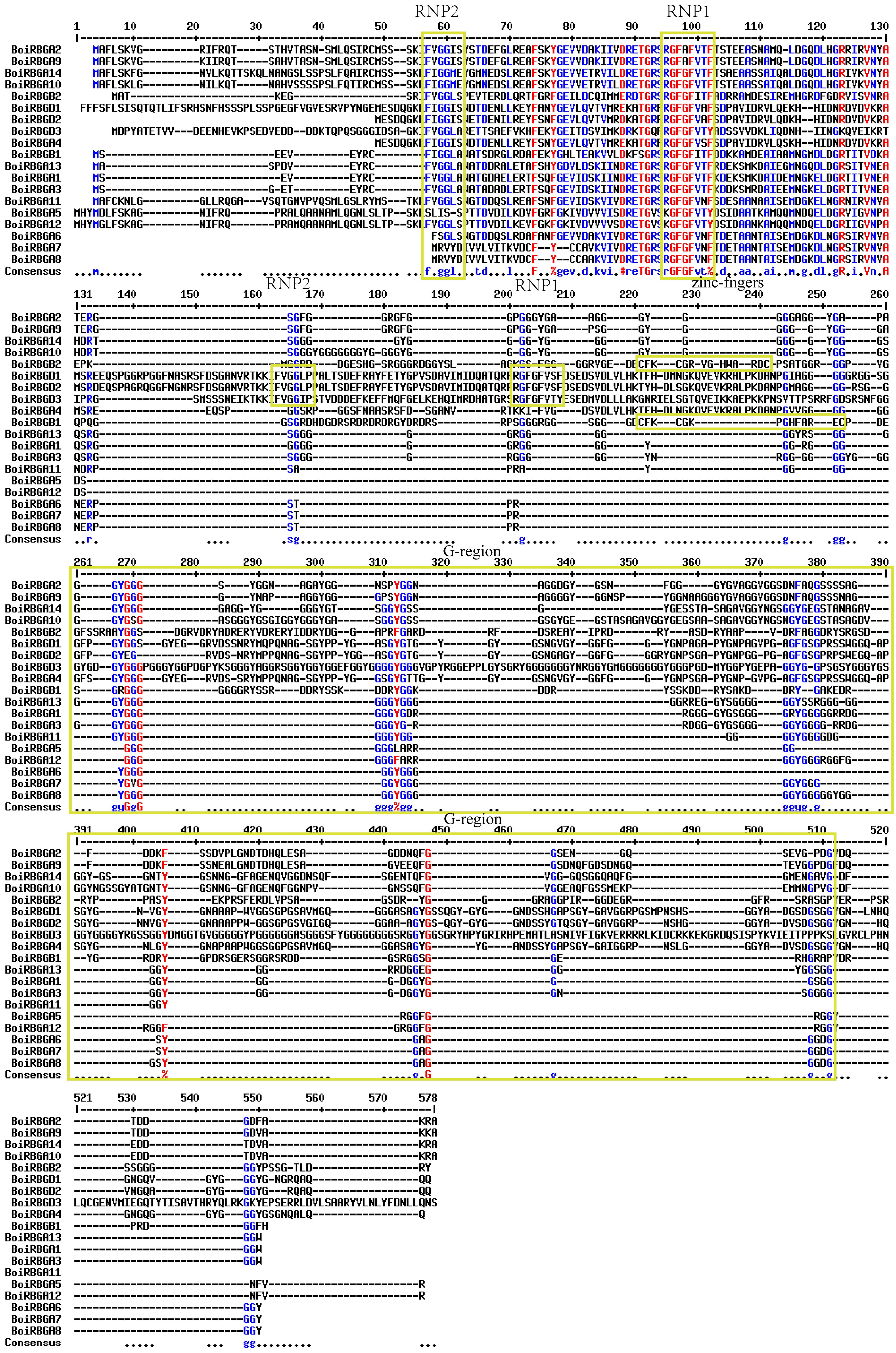

Figure S2. Amino acid sequence alignment of BoiRBGs. The yellow boxes around sequences show conserved motifs, including RRM and glycine-rich domains.
